# Surveillance cardiopulmonary exercise testing can risk-stratify childhood cancer survivors: underlying pathophysiology of poor exercise performance and possible room for improvement

**DOI:** 10.1186/s40959-023-00193-y

**Published:** 2023-11-17

**Authors:** Takeshi Tsuda, Kimberly Davidow, Gina D’Aloisio, Joanne Quillen

**Affiliations:** 1 Nemours Cardiac Center, Nemours Children’s Health, 1600 Rockland Rd, Wilmington, DE 19803 USA; 2 Nemours Center for Cancer and Blood Disorders, Nemours Children’s Health, Wilmington, DE 19803 USA; 3 Department of Pediatrics, Sidney Kimmel Medical College at Thomas Jefferson University, Philadelphia, PA 19107 USA

**Keywords:** Cardiotoxicity, Chemotherapy, Anthracycline, Biomarker, Risk-stratification, Cardiovascular health

## Abstract

**Background:**

Asymptomatic childhood cancer survivors (CCS) frequently show decreased exercise performance. Poor exercise performance may indicate impaired future cardiovascular health.

**Methods:**

Cardiopulmonary exercise testing (CPET) was performed in asymptomatic off-treatment CCS (age ≥ 10 years). Patients were divided into Normal and Poor performance groups by %predicted maximum VO2 at 80%. Both peak and submaximal CPET values were analyzed.

**Results:**

Thirty-eight males (19 Normal, 19 Poor) and 40 females (18 Normal, 22 Poor) were studied. Total anthracycline dosage was comparable among 4 groups. The body mass index (BMI), although normal, and weight were significantly higher in Poor groups. Peak heart rate (HR) and peak respiratory exchange ratio (RER) were comparable in all four groups. Peak work rate (pWR)/kg, peak oxygen consumption (pVO2)/kg, peak oxygen pulse (pOP)/kg, and ventilatory anaerobic threshold (VAT)/kg were significantly lower, whereas heart rate (HR) increase by WR/kg (ΔHR/Δ[WR/kg] was significantly higher in Poor groups. Simultaneously plotting of weight & pVO2 and ΔHR/ΔWR & ΔVO2/ΔHR revealed a distinct difference between the Normal and Poor groups in both sexes, suggesting decreased skeletal muscle mass and decreased stroke volume reserve, respectively, in Poor CCS. The relationship between VAT and pVO2 was almost identical between the two groups in both sexes. Ventilatory efficiency was mildly diminished in the Poor groups.

**Conclusions:**

Decreased skeletal muscle mass, decreased stroke volume reserve, and slightly decreased ventilatory efficiency characterize Poor CCS in both sexes. This unique combined CPET analysis provides useful clinical biomarkers to screen subclinical cardiovascular abnormality in CCS and identifies an area for improvement.

## Background

Recent remarkable progress in cancer treatment has enabled nearly 85% of childhood cancer survivors (CCS) to live beyond 5 years after diagnosis [1]. Consequently, CCS develop varying degrees of long-term adverse health-related problems including relapse of primary malignancy, secondary malignancy, and cardiovascular complications [1–4]. Among those who survive their malignancy, cardiovascular disease is the third leading cause of morbidity and mortality following relapse of primary malignancy and occurrence of secondary cancer [5]. Late cardiovascular complications are frequently insidious, progressive, pervasive, and irreversible. Early identification of high-risk candidates for cardiovascular diseases and initiation of appropriate management for asymptomatic CCS are essential for long-term cardiovascular health and prognosis [6].

To screen for cancer treatment-induced subclinical myocardial impairment, conventional echocardiographic surveillance has been recommended by multiple clinical guidelines to screen at-risk patients [7, 8], but its reliability in detecting subtle subclinical myocardial impairment is limited. Multiple efforts have been made to recognize this subclinical cardiotoxicity, especially with advanced echocardiographic imaging and cardiac magnetic resonance imaging (CMRI), but the results are not consistent. A reliable clinical biomarker has not been identified to detect an early stage of slowly progressive cardiovascular abnormalities in CCS.

Cardiopulmonary exercise testing (CPET) is a useful, noninvasive method to assess cardiopulmonary fitness level in children and adolescents with heart disease [9, 10]. Several studies have demonstrated that CCS have a significantly reduced exercise performance compared with their age-matched peers [11–14]. The underlying mechanisms of reduced exercise performance in these patients are complex and multifactorial and are induced by treatment-mediated systemic cytotoxicity in multiple organs. Poor exercise performance in CCS stems not only from direct myocardial impairment but also from musculoskeletal abnormalities, vascular dysfunction, endocrinopathies, neuropathies of peripheral and central nervous systems, and abnormal pulmonary function [3, 15]. A recent study suggested a genetic predisposition to the development of cancer treatment-induced cardiac dysfunction [16].

In this study, we investigated whether certain CPET parameters can serve as reliable clinical biomarkers to identify subclinical cardiovascular abnormalities. We hypothesized that our novel CPET approach in combining peak and submaximal parameters (“two-dimensional analysis” [17, 18]) can delineate possible underlying mechanisms of poor exercise performance in CCS.

## Methods

A retrospective chart review of CPET data from the database of the Exercise Laboratory, Nemours Cardiac Center, at Nemours Children’s Health in Wilmington, DE, was conducted from 2018 to 2021. The study was approved by the Nemours Institutional Review Board.

### Patients

We retrospectively studied asymptomatic CCS followed at the Cancer Survivorship Program, Nemours Center for Cancer and Blood disorders, at Nemours Children’s Health in Wilmington, DE, who were referred for CPET to assess physical fitness levels. Inclusion criteria were (1) age ≥ 10 years, (2) off cancer treatment ≥ 1 year, (3) intact musculoskeletal system and neurological function, (4) body mass index (BMI) < 30 kg/m^2^, and (5) left ventricular shortening fraction (LVSF) ≥ 28% or left ventricular ejection fraction (LVEF) ≥ 55% by echocardiography. Age, sex, height, weight, and BMI of the patients were collected at the time of CPET. Primary diagnosis, age at diagnosis, cumulated dosage of anthracycline (mg/m^2^), and history of radiation therapy were recorded.

### Cardiopulmonary exercise testing

The study was performed on bike ergometer (VIA Sprint 150 P, Yorba Linda, CA) following RAMP (Raise, Activate, Mobilize, and Potentiate) protocol (approximately 0.3 W/kg/min increment). In addition to vital signs and continuous ECG monitoring, oxygen consumption (VO2), carbon dioxide production (VCO2), and minute ventilation (VE) were continuously measured. The exercise protocol was continued until the patient stopped due to symptomatic limitations. Achievement of peak exercise level was confirmed by either peak heart rate (HR) of more than 90% of estimated maximum HR for age (220 – age [yrs]) or respiratory exchange ratio (RER) of 1.1 or higher. Those who did not reach a peak exercise were not enrolled in this study.

Peak and submaximal exercise parameters were obtained. Peak values of HR (pHR; beats/min), systolic blood pressure (SBP; mmHg), work rate (pWR; W), VO2 (pVO2; L/min), oxygen pulse (pOP; ml/beat), minute ventilation (pVE; L/min), and peak RER (pRER: pVCO2/pVO2) were measured. Peak VO2 was also presented by %predicted maximum VO2 (PmaxVO2). Predicted maxVO2 was obtained from the following formula [19];

$$\begin{aligned} & {\text{PmaxVO2}}\,({\text{ml/kg/min}})  \\ & \quad = 37.9022 - [0.1957*{\text{Age}}\, ({\text{years}})] \\ & \quad \quad + 3.3287*{\text{Gender}}. \\ & {\text{Gender; Male 1, Female 0}}.\end{aligned}$$

Submaximal CPET parameters consist of ventilatory anaerobic threshold (VAT; L/min) and submaximal slope parameters, including ΔVO2/ΔHR (a surrogate of stroke volume), ΔHR/ΔWR (heart rate dependency), ΔVE/ΔVCO2 (an inverse of ventilatory efficiency), and ΔVO2/ΔWR (oxygen uptake per work or “oxygen cost”). These submaximal slope parameters represent trends up to anaerobic threshold (AT). All CPET parameters were presented as absolute values. Some parameters were also indexed by body weight.

### Subgrouping

We divided CCS into four subgroups by PmaxVO2 in both male and female CCS. Normal and Poor groups were defined as pmaxVO2 ≥ 80% and < 80%, respectively.

### Statistics

Distribution of patients’ demographics as well as peak and submaximal parameters were compared between the subgroups. The data were shown as mean ± standard deviation (SD) for continuous variables, unless otherwise notified. Count and percentage for categorical variables were reported. Two-sample t-test and chi-square test were used to compare the mean and proportion, respectively, between the two groups. Model/test assumptions were checked before data analysis. All tests were two-tailed at the level of significance of 0.05.

## Results

From January 2018 to December 2021, we reviewed data of 38 male and 40 female CCS in this study who met the inclusion criteria. The patients were divided into Normal exercise performers (%pmaxVO2 ≥ 80%) and Poor performers (%pmaxVO2 < 80%), as defined.

### Normal and poor exercise performers

There were 38 Normal performers (20 males and 18 females) and 40 Poor performers (18 males and 22 females) (Table 1). The age at diagnosis of malignancy showed no difference in males but was significantly higher in Poor group than in Normal group for females. There was no significant difference in years after diagnosis between the two groups in either sex. The distribution of primary disease was not different between Normal and Poor groups in either sex. Total cumulated anthracycline dosage and incidence of chest radiation were comparable between Normal and Poor groups in both sexes. There was no difference in left ventricular systolic function (left ventricular shortening fraction [%LVSF] and ejection fraction [%LVEF]) by echocardiogram among all four groups.

**Table 1:** Background Information of Childhood Cancer Survivors

	Male				Female		
PmaxVO2	Normal (≥ 80%)		Poor (< 80%)	p value	Normal (≥ 80%)	Poor (< 80%)	p value
Number	20		18		18	22	
Age at Diagnosis (years)	7.1 ± 4.4		9.1 ± 5.4	0.24	4 ± 2.9	7.3 ± 4.8	**0.018**
Years after Diagnosis	6.8 ± 3.8		6.3 ± 4.2	0.75	10.3 ± 3.7	7.8 ± 5.0	0.09
**Diagnosis**							
**Leukemia**	8 (40%)		11 (61%)	0.79	6 (33%)	6 (27%)	0.91
ALL	5		8		5	5	
AML	1		0		0	1	
Post-HSCT	2		3		1	0	
**Lymphoma**	8 (40%)		4 (22%)	0.70	0 (0%)	6 (27%)	0.60
Hodgkin	3		1		0	5	
Non-Hodgkin	5		3		0	1	
**Solid Tumor**	4 (20%)		3 (17%)	0.94	12 (67%)	10 (46%)	0.76
Wilms’ tumor	1		0		3	5	
Osteosarcoma	0		1		0	2	
Ewing sarcoma	0		0		3	1	
Neuroblastoma	0		1		4	1	
Other	3		1		2	1	
**Treatment**							
**Anthracycline (mg/m** ^**2**^ **)**	183 ± 102		164 ± 90	0.57	219 ± 122	218 ± 112	0.98
**Chest RT**	1 (5%)		3 (2%)	0.32	7 (39%)	9 (41%)	0.62
≥ 15 Gy	0		1		5	6	
< 15 Gy	1		2		2	3	
**Echocardiogram**							
LVSF (%)	35.0 ± 4.6		34.7 ± 3.0	0.86	33.8 ± 3.8	34.7 ± 2.4	0.34
LVEF (%)	63.6 ± 4.9		61.5 ± 4.5	0.21	58.7 ± 3.9	61.3 ± 3.5	0.05

%pmaxVO2: %predicted maximum oxygen consumption, ALL: acute lymphoblastic leukemia, AML: acute myeloblastic leukemia, Post-HSCT: post hematopoietic cell transplant, RT: radiation therapy, LVSF: left ventricular shortening fraction, LVEF: left ventricular ejection fraction

### Peak and submaximal CPET parameters

The results of CPET are presented in Table 2. There is no significant difference in age at CPET in all four groups. Weight and BMI, although normal, were significantly higher in Poor groups than in Normal groups in both sexes. There was no significant difference in pHR or pRER. Normal groups showed significantly higher peak weight-indexed CPET values including WR/kg, pVO2/kg, and pOP/kg than Poor groups in both sexes, consistent with our definition of grouping. For submaximal parameters, Poor groups exhibited significantly lower VAT/kg than Normal groups in both sexes. Δ[VO2/kg]/ΔHR, a surrogate parameter for stroke volume, was significantly lower and ΔHR/Δ[WR/kg], heart rate dependency, was significantly higher in Poor groups than in Normal groups in both sexes, suggesting limited stroke volume reserve resulting in faster exercise-induced HR increase in Poor groups. Ventilatory efficiency (ΔVE/ΔVCO2) was comparable in all four groups. ΔVO2/ΔWR, oxygen cost or oxygen uptake per work, was significantly lower in Poor group than in Normal group only in females.

**Table 2:** Peak and Submaximal CPET Parameters

	Male			Female			
%pmaxVO2	Normal (≥ 80%)	Poor (< 80%)	p value		Normal (≥ 80%)	Poor (< 80%)	p value
Number	20	18			18	22	
Age at CPET (years)	13.9 ± 2.3	15.4 ± 3.2	0.10		14.3 ± 2.6	15.1 ± 3.2	0.36
Weight (kg)	52.2 ± 13.7	66.0 ± 15.8	**0.01**		51.6 ± 14.4	59.8 ± 11.5	**0.03**
Height (cm)	162.4 ± 14.1	166.5 ± 13.6	0.81		156.4 ± 13.0	159.2 ± 8.3	0.27
BMI (kg/m^2^)	19.5 ± 3.1	23.6 ± 3.9	**0.0006**		20.7 ± 3.5	23.6 ± 4.5	**0.02**
**Peak CPET Parameters**							
pHR (beats/min)	189 ± 8	188 ± 14	0.92		187 ± 5	189 ± 8	0.70
pSBP (mmHg)	148 ± 24	147 ± 20	0.82		141 ± 20	149 ± 22	0.85
pWR (W)	146 ± 44	128 ± 48	0.24		110 ± 41	97 ± 28	0.22
pWR/kg (W/kg)	2.73 ± 0.39	1.92 ± 0.43	**< 0.0001**		2.02 ± 0.40	1.62 ± 0.39	**< 0.0001**
pVO2 (L/min)	2.23 ± 0.53	1.89 ± 0.53	0.064		1.79 ± 0.52	1.42 ± 0.30	**0.007**
pVO2/kg (ml/kg/min)	42.5 ± 5.2	29.1 ± 4.9	**< 0.0001**		36.4 ± 7.0	24.0 ± 4.7	**< 0.0001**
%PmaxVO2 (%)	94.4 ± 11.4	64.0 ± 10.3	**< 0.0001**		102 ± 18	64.7 ± 11.1	**< 0.0001**
pOP (ml)	12.2 ± 3.0	10.1 ± 2.9	**0.04**		9.4 ± 2.5	7.7 ± 1.7	**0.02**
pOP/kg (ml/kg)	0.23 ± 0.03	0.16 ± 0.03	**< 0.0001**		0.19 ± 0.03	0.13 ± 0.03	**< 0.0001**
pVE (L/min)	81.9 ± 21.4	77.5 ± 20.3	0.54		64.3 ± 15.7	57.2 ± 17.8	0.20
pRER	1.20 ± 0.08	1.28 ± 0.16	0.092		1.21 ± 0.09	1.26 ± 0.13	0.18
**Submaximal CPET Parameters**							
VAT (L/min)	1.36 ± 0.35	1.19 ± 0.36	0.15		1.20 ± 0.33	0.99 ± 0.27	**0.03**
VAT/kg (ml/kg/min)	26.6 ± 5.3	18.4 ± 4.5	**< 0.0001**		24.5 ± 5.0	16.8 ± 4.7	**< 0.0001**
Δ[VO2/kg]/ΔHR (ml/kg/min)	0.36 ± 0.07	0.25 ± 0.07	**< 0.0001**		0.32 ± 0.10	0.19 ± 0.07	**< 0.0001**
ΔHR/Δ[WR/kg] (kg/W)	31.6 ± 7.6	39.6 ± 11.8	**0.02**		36.2 ± 9.2	47.9 ± 12.9	**0.003**
ΔVE/ΔVCO2	23.5 ± 1.9	24.2 ± 4.1	0.52		24.7 ± 3.0	25.0 ± 3.2	0.73
ΔVO2/ΔWR (L/min/W)	11.3 ± 1.7	9.5 ± 3.8	0.11		12.4 ± 4.8	8.9 ± 7.6	**0.006**

pVO2: peak oxygen consumption, pmaxVO2: predicted maximum oxygen consumption, CPET: cardiopulmonary exercise testing, BMI: body mass index, pHR: heat heart rate, pSBP: peak systolic blood pressure, W: watt, pWR: peak work rate, pOP: peak oxygen pulse, pVE: peak minute ventilation, pRER: peak respiratory exchange ratio, VAT: ventilatory anaerobic threshold, WR: work rate. VCO2: carbon dioxide production

### Two-dimensional CPET analysis

Simultaneous assessment of two CPET parameters between Normal and Poor exercise performers provided a more mechanistic interpretation of compromised exercise capacity in CCS.

**Fig. 1:**
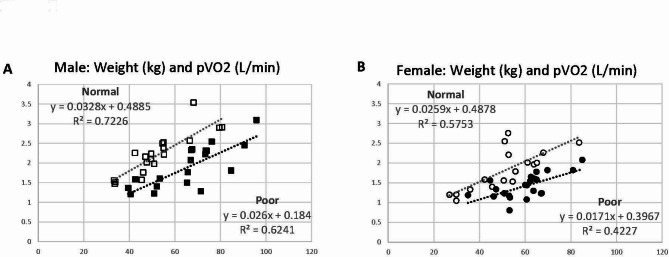
Scatter graphs with weight (kg) in x-axis and peak oxygen consumption (pVO2: L/min) in y-axis to characterize the skeletal muscle effects in childhood cancer survivors (CCS) (**A**) Males, and (**B**) Females. Normal and Poor exercise performers are defined as %pmaxVO2 ≥ 80% and pVO2 < 80% in both sexes, respectively. (**A**) Open square: Normal males; Closed square: Poor males. (**B**) Open circle: Normal females; Closed circle: Poor females. Normal performers tended to show higher pVO2 with a given weight in both sexes than Poor performers, suggesting better skeletal muscle effects (skeletal muscle mass and possibly aerobic energy metabolism) in Normal performers

*Skeletal Muscle Effects*. Weight and pVO2 were plotted for the x- and y-axis, respectively (Fig. 1). Both Normal and Poor groups showed excellent positive linear relationships (R^2^ = 0.42 to 0.72), but there was a marked difference between the Normal and Poor groups: Poor groups revealed significantly lower pVO2 at a given weight, suggesting decreased metabolically active muscle mass and possibly oxygen uptake capacity (= skeletal muscle effects) per weight in the Poor groups. This trend difference between Normal and Poor performers was equally seen in male and female CCS.

**Fig. 2:**
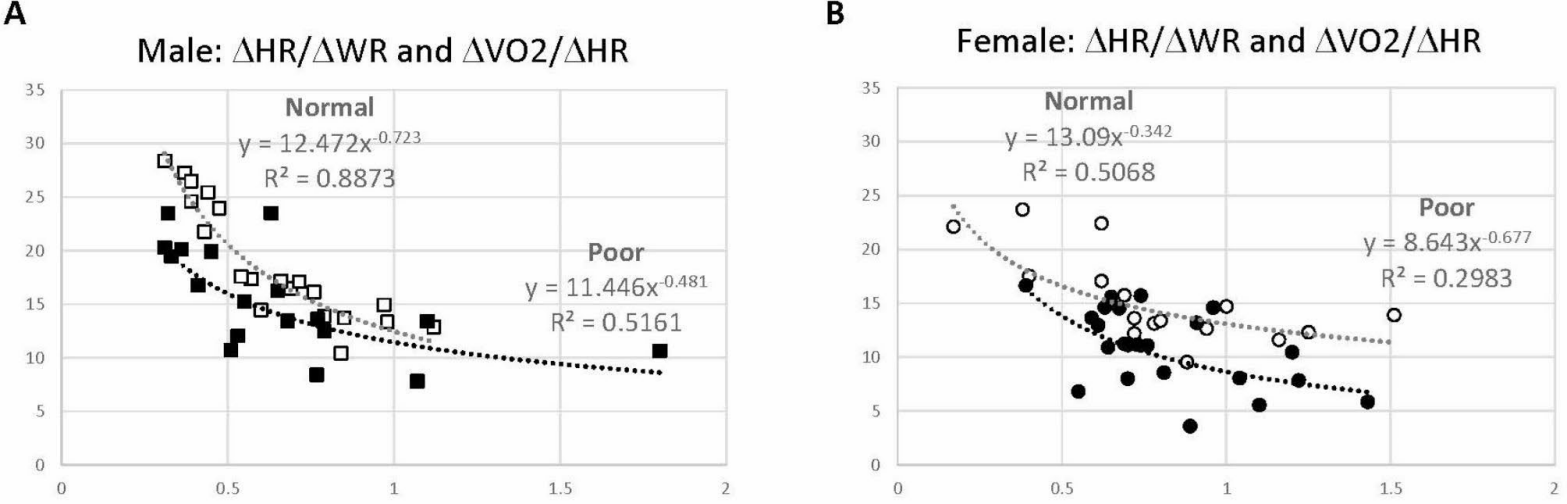
Scatter graphs with ΔHR/ΔWR (heart rate dependency) in x-axis and ΔVO2/ΔHR (surrogate of stroke volume) in y-axis to characterize the stroke volume reserve in CCS. (**A**) Males, and (**B**) Females. Normal performers tend to show higher ΔVO2/ΔHR and lower ΔHR/ΔWR compared with Poor performers in both sexes, suggesting higher stroke volume reserve in Normal performers

*Stroke Volume Reserve*. The ΔHR/ΔWR and ΔVO2/ΔHR were plotted for the x- and y-axis, respectively (Fig. 2). The ΔHR/ΔWR represents HR increase in response to a given WR (indicating HR dependency), whereas ΔVO2/ΔHR signifies a surrogate of stroke volume during submaximal exercise. As ΔHR/ΔWR increases, ΔVO2/ΔHR decays exponentially. Higher HR dependency tends to be associated with lower stroke volume and vice versa. There is a noticeable difference between Normal and Poor groups in both males and females; Poor performers tend to show lower ΔVO2/ΔHR in combination with higher ΔHR/ΔWR in both males and females, suggesting their decreased stroke volume reserve.

**Fig. 3:**
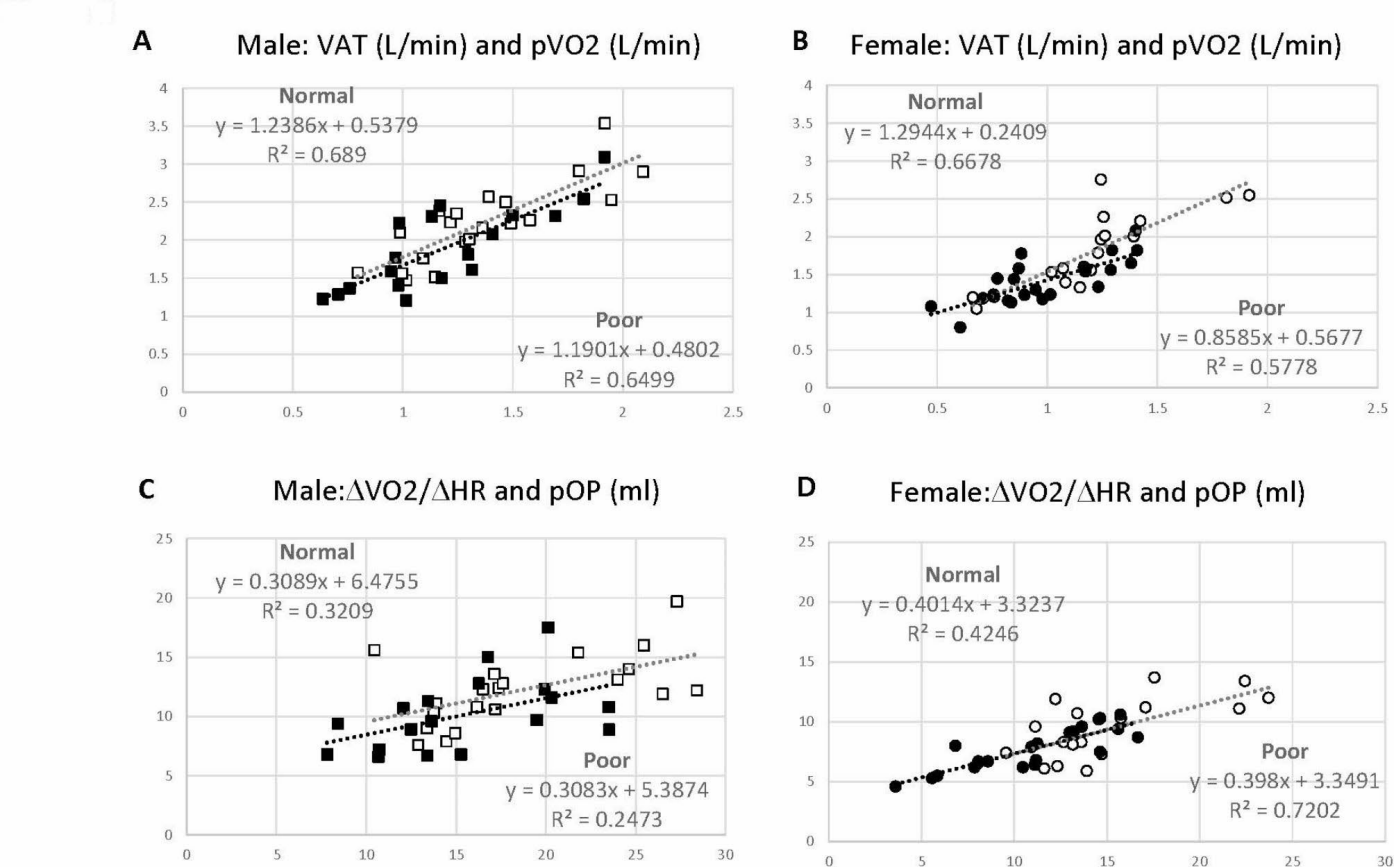
**A** and **B**: Scatter graphs with ventilatory anaerobic threshold (VAT) in x-axis and peak oxygen consumption (pVO2) in y-axis to assess exercise endurance beyond anaerobic threshold (AT) when anaerobic metabolism is supposed to start. **C** and **D**: Scatter graphs with ΔVO2/ΔHR (surrogate of stroke volume) in x-axis and peak oxygen pulse (pOP: stroke volume surrogate at the peak exercise) in y-axis

*Exercise Endurance beyond Anaerobic Threshold (AT)*. The relationship between VAT and pVO2 was studied (Fig. 3A and **B**). There are excellent positive correlations between VAT and pVO2 in both males (R^2^: 0.65 to 0.69) and females (R^2^: 0.58 to 0.67). The regression lines are almost identical between Normal and Poor groups in both sexes, suggesting there is no difference in exercise endurance beyond AT. TheΔVO2/ΔHR and pOP represent stroke volume surrogate during submaximal phase and at the peak exercise, respectively (Fig. 3C and **D**). There are good to excellent correlations between ΔVO2/ΔHR and pOP in both males (R^2^: 0.23 to 0.31) and females (R^2^: 0.42 to 0.72) with no significant difference between Normal and Poor groups, concordant with the relationship between VAT and pVO2. These results suggest that the tolerance to anaerobic metabolism is comparable between Normal and Poor CCS in both sexes.

**Fig. 4:**
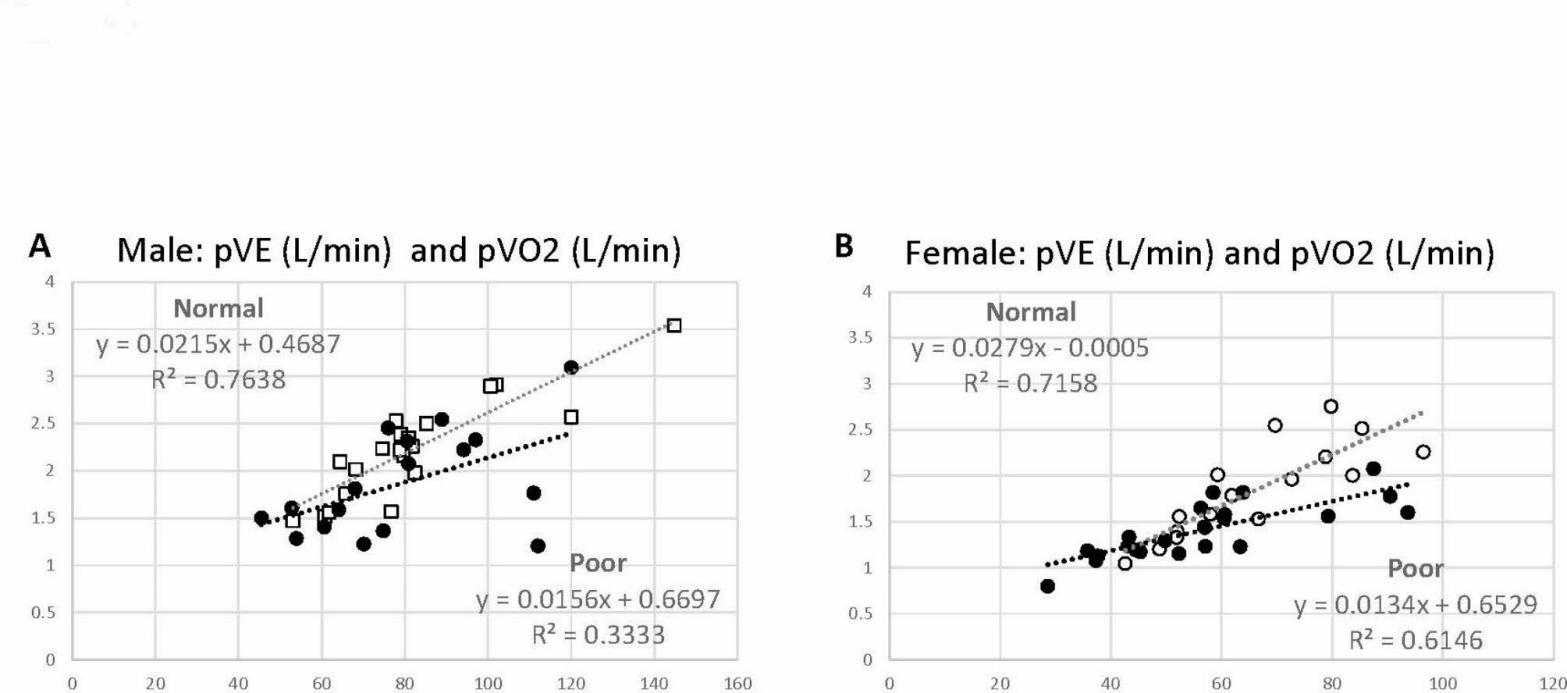
Scatter graphs with peak minute ventilation (pVE) in x-axis and peak oxygen consumption (pVO2) in y-axis to assess the ventilatory efficiency for oxygen uptake in CCS. **A**. Males, and **B**. Females. Normal performers showed slightly better pVO2 with the same pVE, suggesting their slightly better oxygen uptake efficiency than Poor performers in both sexes

*Ventilatory Efficiency for Oxygen Uptake*. Peak VE and pVO2 were plotted for the x- and y-axis, respectively, where a slope indicates the ventilatory efficiency for oxygen uptake (Fig. 4). There is an excellent correlation (R^2^ = 0.61 to 0.76) between pVE and pVO2 in all groups except Poor males (R^2^ = 0.33). There is a slight decline in a slope of the Poor performers compared with the Normal performers in both males and females, suggesting slightly decreased efficiency in oxygen uptake in Poor groups.

## Discussion

Using our unique CPET approach comparing Normal and Poor exercise performers in CCS, we studied the underlying pathophysiology of poor exercise performance in CCS, including (1) skeletal muscle mass effects, (2) stroke volume reserve, (3) exercise endurance beyond VAT, and (4) breathing efficiency. This retrospective CPET analysis helped us delineate possible underlying mechanisms of poor exercise performance in CCS, which can serve as a useful clinical biomarker in identifying subclinical cardiovascular abnormalities in CCS and an area for potential improvement toward better cardiovascular health.

### Screening of subclinical late-onset cardiovascular complications in CCS

Anthracycline-induced cardiotoxicity plays a central role in the development of late cardiovascular complications in CCS that can occur decades after the initial treatment [20–23]. Late-onset cardiotoxicity is insidious and nonspecific yet progressive and irreversible [24]. Thus, early recognition of silent cardiotoxicity is essential to protect patients from developing symptomatic cardiomyopathy or advanced heart failure. Reliability of echocardiography in predicting late cardiovascular complications is limited, as normal echocardiogram at younger ages may not be indicative of freedom from late cardiotoxicity [21, 25]. Long-term cardiovascular complications for CCS pertain not only to direct myocardial dysfunction and heart failure but also include increased incidence of coronary artery disease, stroke, and variable vascular diseases [26]. Multiple efforts have been made to recognize this subclinical stage of cardiotoxicity, especially from noninvasive image modalities including advanced echocardiographic imaging and cardiac magnetic resonance imaging (CMRI). However, late cardiovascular complications in CCS stem not only from direct cardiotoxicity-induced myocardial impairment; they also involve multiple organs that interact the cardiovascular system.

Poor cardiopulmonary fitness level is known to be strongly correlated with higher risk of heart failure, cardiovascular events, and overall mortality in the general population [27, 28] and in cancer survivors [29, 30]. Several studies have indicated that CCS present with significantly reduced exercise capacity compared with the age-match peers, representing poor functionality, quality of life, and health status [12, 14, 18, 31–35]. A detailed systematic approach with peak and submaximal CPET parameters will provide a wealth of physiological information that has been underused [9]. In our 78 CCS, no one showed abnormal echocardiogram, yet half of them exhibited subnormal peak exercise performance. Considering long-term cardiovascular health, this fact should not be overlooked.

### Mechanistic assessment of poor exercise performance by comprehensive CPET analysis

Our unique CPET analysis demonstrated three main mechanisms for decreased exercise performance in CCS. Poor exercise performance in CCS may be attributed to a combination of primary cardiotoxicity (direct myocardial impairment induced by cytotoxic drugs), treatment-mediated adverse effects on other organ systems (skeletal muscle, blood vessels, autonomic nervous system, and lung), and physical deconditioning secondary to their unfavorable lifestyle, as indicated earlier.

The first responsible mechanism is decreased skeletal muscle effects, probably due to quantitatively decreased muscle mass and altered aerobic metabolism, as shown in Fig. 1. Poor performers presented significantly lower pVO2 values than Normal performers at the same weight in both sexes, suggesting decreased muscle mass in Poor CCS as pVO2 is closely corelated with skeletal muscle mass [36]. Sarcopenia is characterized by low muscle quantity, high fat accumulation in the muscle, low muscle strength, and low physical performance [37]. The chemotherapy-induced delayed skeletal muscle dysfunction is probably not fully reversible, and impairment of satellite cells, muscle motor innervation, or mitochondrial function may be responsible for impaired aerobic metabolism [38]. Skeletal myopathy in CCS is likely induced by mitochondrial dysfunction resulting in exacerbation of cell death and loss of regenerating capacity, which may be responsible for fatigue, muscle wasting, impaired regenerative capacity, and exercise intolerance [39]. Excessive fat mass in combination with quantitively decreased skeletal muscle mass and myopathic changes further deteriorate exercise performance in CCS.

Second, compromised stroke volume reserve is likely another reason for low exercise performance in Poor groups, as indicated by significantly lower pOP/kg, lower Δ[VO2/kg]/ΔHR, and higher ΔHR/Δ[WR/kg] in Poor groups (Table 2). In response to an incremental exercise protocol, faster HR increase usually indicates lower stroke volume reserve. This was also supported by the relationship between ΔHR/ΔWR and ΔVO2/ΔHR (Fig. 2). Our data are concordant with the report by Foulkes et al., who studied CMRI at rest and at peak exercise in 20 CCS from 8 to 24 years of age and demonstrated that a decreased exercise capacity is associated with impaired hemodynamics (= decreased cardiac index and decreased peripheral oxygen extraction) and systolic functional reserve (= reduced LVEF increase) measured during exercise [40].

A third possible contributing factor for decreased exercise performance is inefficient breathing pattern with decreased oxygen utilization, as shown in Fig. 4. As ΔVE/ΔVCO2 was comparable between Normal and Poor groups, there may not be a significant difference in major ventilatory mechanics. As there was no notable drop of oxygen saturation at peak exercise in our cohort (data not shown), there should not be any relevant difference in oxygenation. Adult cancer patients have been shown to have relatively reduced respiratory muscle strength and lung diffusion capacity, which are likely responsible for a rapid and shallow breathing pattern during exercise [41]. It is not entirely clear, however, how these altered lung mechanics result in reduced oxygen uptake in relation to VE.

### Treatable vs. untreatable conditions after cancer treatment

It is frequently challenging to identify subclinical changes of cardiovascular system in CCS, including unrecognizable insidious deterioration by sedentary lifestyle and a subtle improvement by routine exercise training. Importantly, the causes of poor exercise performance in CCS are not merely due to cardiotoxicity-induced direct myocardial impairment but also to secondary physical deconditioning. Some conditions can be improved by exercise training, whereas others may not be altered due to their irreversible nature.

We demonstrated certain responsible mechanisms for decreased exercise performance other than direct cardiotoxicity: decreased skeletal muscle effects (Fig. 1) and an inefficient breathing technique, in part, due to reduced respiratory muscle strength (Fig. 4). Anthracycline-induced direct cardiotoxicity has been extensively studied, but there has been no known effective treatment to reverse the processes of cellular injury, including increased reactive oxygen species (ROS) and increased intracellular calcium overload, suppression of protein synthesis, mitochondrial dysfunction and alteration in cardiac energy metabolism, impaired DNA-replication, and chronic inflammation [42–48]. Thus, it is reasonable to emphasize the improvement of treatable conditions when introducing a cardiac rehabilitation program; improving skeletal muscle integrity is an important therapeutic goal to sustain overall health resilience in CCS in addition to routine aerobic exercise. Our unique CPET methods are simple and useful in identifying subclinical cardiovascular abnormalities and recognizing the improvement of skeletal muscle effects and breathing efficiency with exercise training in CCS.

This study has several limitations. First, our patients are a heterogenous population with variable physical conditioning, body habitus, level of puberty, and ethnic background; all affect exercise performance assessed by CPET. There were different types of malignancy and cancer treatment in our cohort, which may affect differently the performance of multiple organs involved in exercise capacity. In addition, there may be a selection bias at the patient enrollment, as not all CCS participated in CPET. Second, although we perceived pOP and ΔVO2/ΔHR as surrogate markers for stroke volume both as absolute and weight-indexed value, it is not altogether accurate as peripheral oxygen extraction was not examined in this study. Third, obese patients (BMI > 30 kg/m^2^) were not included in the study primarily to secure valid CPET analysis. However, obesity may be one important pathological feature of CCS after cancer treatment, which could be contributing to the development of future cardiovascular complications. Fourth, we did not measure lean body mass in this study. Having lean body mass measurement would have significantly enhanced our argument. Lastly, this is a retrospective study with a relatively small cohort in a single institution. A future prospective, multicenter study may eliminate the selection bias and will provide statistical power to validate our hypothesis.

## Conclusions

Our current CPET analysis addressed three responsible mechanisms of poor exercise performance in CCS, including impaired skeletal muscle performance, reduced stroke volume reserve, and breathing inefficiency. By targeting treatable conditions, including skeletal muscle conditioning and breathing techniques through an exercise training program in combination with nutritional management to reduce excessive body fat, we may be able to introduce beneficial effects on patients’ daily functionality, future quality of life, health span, and survival. These CPET parameters serve as excellent clinical biomarkers in identifying silent cardiovascular abnormalities in asymptomatic CCS and in assessing improvement of cardiovascular reserve and physical conditioning through exercise.

## Data Availability

Yes.
